# Enhanced Thermoelectric Properties of Phosphorene via Quantum Size Effects and Relaxation Time Tuning

**DOI:** 10.3390/ma18112506

**Published:** 2025-05-26

**Authors:** Zhiqian Sun, Chenkai Zhang, Guixian Ge, Gui Yang, Jueming Yang

**Affiliations:** 1 State Key Laboratory of Advanced Energy Storage Materials and Technology, College of Sciences, Shihezi University, Shihezi 832000, Chinaguixiange@shzu.edu.cn (G.G.); 2 School of Mechanical and Electrical Engineering, Chuzhou University, Chuzhou 239000, China

**Keywords:** phosphorene, band structures, thermoelectric property, density functional theory

## Abstract

Black phosphorus is a promising thermoelectric (TE) material because of its high Seebeck coefficient and high electrical conductivity. In this work, the TE performance of bulk black phosphorus and single-layer phosphorene under uniaxial strain is studied using first-principles calculations and Boltzmann transport theory. The results show relatively excellent TE performance along the armchair direction for both black phosphorus and phosphorene in our study. However, high lattice thermal conductivity is the key adverse factor for further enhancing the TE performance of phosphorus. The *ZT* value can only reach up to 0.97 and 0.73 for *n*- and *p*-type black phosphorus at 700 K, respectively. Owing to quantum size effects, black phosphorene has lower lattice thermal conductivity than black phosphorus. At the same time, two-dimensional (2D) phosphorene exhibits increased electronic energy compared with bulk black phosphorus, resulting in a larger bandgap and reduced electrical conductivity due to the quantum confinement effect. Thus, the TE performance of *n*-type phosphorene can be partially improved, and the *ZT* value reaches up to 1.41 at 700 K. However, the *ZT* value decreases from 0.73 to 0.70 for *p*-type phosphorene compared with bulk phosphorus at 700 K. To further improve the TE performance of phosphorene, a tensile strain is applied along the armchair direction. Subsequent work indicates that uniaxial strain can further optimize phosphorene’s TE properties by tuning hole relaxation time to improve electrical conductivity. Strikingly, the *ZT* values exceed 1.7 for both *n*- and *p*-type phosphorene under 4.5% tensile strain along the armchair direction at 700 K because of increased electrical conductivity and decreased lattice thermal conductivity.

## 1. Introduction

TE materials can realize heat-electricity conversion directly [1,2,3]. The TE performance is described [4,5] by a figure of merit *ZT* = *S*^2^*σT/*(*k_tot_*). Here, *S* is the Seebeck coefficient; *σ* is the electrical conductivity; *k_tot_* is the total thermal conductivity, which is the sum of the electronic thermal conductivity *k_e_* and lattice thermal conductivity *k_l_* (*k_tot_* = *k_e_* + *k_l_*); and *T* is the absolute temperature. To achieve high *ZT* values, high electrical conductivity and low thermal conductivity are both needed [6,7]. To date, various approaches have been developed to simultaneously optimize the electronic and thermal transport properties of TE materials to enhanced TE performance. These include band structure engineering [7,8,9] to enhance the power factor (*S*^2^*σ*), enhancing phonon scattering [10] to reduce thermal conductivity (*k_tot_*), and the exploration of materials with low thermal conductivity [11,12]. Based on these strategies, remarkable achievements have been made in numerous material systems, and several problems have severely prohibited the practical application of conventional TE materials (Bi_2_Te_3_/Sb_2_Te_3_ superlattice (*ZT* ≈ 2.4, T = 300 K) [13], CoSb_3_ (*ZT* > 1.2, T = 800 K) [14], AgPb_m_SbTe_2+m_ (*ZT* ≈ 2.2, T = 800 K) [15], PbTe-SrTe (*ZT* ≈ 2.2, T = 915 K) [10], etc.). Although these TE materials have high TE performance, the existence of toxic, rare, or expensive constituent elements has severely hampered their practical applications as TE devices. However, black phosphorus is non-toxic and abundantly available in the Earth’s crust compared with the conventional TE materials. The space group of black phosphorus is *Cmca*, and it possesses a layered, puckered honeycomb structure, with individual layers held together by Van der Waals forces. Moreover, previous studies have found that it has higher electrical conductivity and lower lattice thermal conductivity along the armchair direction than that along the zigzag direction, suggesting it is maybe a promising TE candidate [16,17].

In 2014, Lv et al. calculated the electronic structure of black phosphorus and phosphorene (obtained by exfoliating bulk black phosphorus) using the Vienna ab initio software package (VASP) and comparatively studied the TE performance of black phosphorus and phosphorene using the Boltzmann transport theory [18]. They reported that the *ZT* values of black phosphorus and phosphorene were 0.22 and 0.30, respectively, at 300 K. However, Lv et al. did not calculate the lattice thermal conductivity. Moreover, they set the Lorenz number *L* as 1.5 × 10^−8^ WΩ/K^2^, which is small for black phosphorus, as can be seen from the work by Zhang et al. [19] in 2016, Zhang et al. studied the TE performance of black phosphorus. They found the *ZT* value of black phosphorus could reach up to 1.1 at 800 K. However, the lattice thermal conductivity (155.7 W/mK and 65.1 W/mK along the zigzag and armchair directions, respectively) they calculated is obviously higher than the experimental results (86 W/mK and 34 W/mK along the zigzag and armchair directions, respectively) [20]. In 2019, Duan et al. [21] found group VA-doped black phosphorus exhibited excellent TE performance: the ZT values of NP_7_, SbP_7_, and BiP_7_ can exceed 2 at 800 K. These theoretical studies primarily focused on doping strategies to enhance black phosphorus’ TE performance. Here, we propose an alternative approach by exploring low-dimensional systems (monolayer phosphorene) and applying uniaxial strain to optimize its TE properties. Although the bulk XP_7_ (X = N, Sb, Bi) phase has not yet been unambiguously demonstrated in experiments, the monolayer phosphorene has been successfully synthesized experimentally [22,23]. In the previous studies, they did not precisely calculate the lattice thermal conductivity due to the limitations of the available technology at that time. However, to obtain more reasonable *ZT* values of black phosphorus and phosphorene, the reliable calculation of lattice thermal conductivity is necessary. Therefore, the lattice thermal conductivity is also precisely calculated apart from the Seebeck coefficient, electrical conductivity, and electronic thermal conductivity here.

In this study, the crystal and electronic structure, phonon and electronic transport, and TE properties of black phosphorus and phosphorene under uniaxial strain are studied using first-principles calculations, Boltzmann transport theory, and relaxation time approximation. The *ZT* value of phosphorene can reach up to 1.41 along the armchair direction at 700 K. This *ZT* value is improved by nearly 50% compared with black phosphorus, suggesting that exploring low-dimensional black phosphorus is an effective way to enhance its TE performance. Subsequent work indicates that TE performance of phosphorene can be further optimized by engineering the relaxation time of holes to enhance electrical conductivity by employing uniaxial strain. Strikingly, the *ZT* values for both *p*- and *n*-type phosphorene exceed 1.7 under 4.5% tensile strain along the armchair direction at 700 K. This result demonstrates that the TE performance of phosphorene can be enhanced by engineering relaxation time.

## 2. Computational Details

In this section, we use VASP software, version 5.3, [24] to optimize bulk black phosphorus and 2D phosphorene. During the optimization process, the Perdew–Burke–Ernzerhof (PBE) [25] exchange correlation function under the generalized gradient approximation (GGA) [26] is adopted. The plane-wave cutoff energy is set to 400 eV. The structure is considered converged when the energy difference between two consecutive steps is less than 10^−6^ eV and the Hellmann–Feynman forces are less than 0.01 eV/Å. Different Monkhorst–Pack k-point meshes are employed for black phosphorus and phosphorene during structural relaxation. Specifically, a k-point grid of 19 × 6 × 14 is used for black phosphorus, and a grid of 18 × 13 × 1 is adopted for phosphorene to optimize their crystal structures. The BoltzTrap, version 1.2.5, [27] package, based on the semi-classical Boltzmann transport theory combined with the relaxation time approximation, is used to predict the electronic transport properties of the materials based on the band structure calculated from the first-principles theory.

Here, the deformation potential theory is used to estimate the carrier mobility to further obtain the electron relaxation time. For three-dimensional (3D) materials, the following formula is used to estimate the relaxation time [28]:

(1) $$\mu^{3D}=\frac{2\sqrt{2}\pi e\hbar^{4}C^{3D}}{3(k_{B}T{)}^{3/2}{\left|m^{*}\right|}^{5/2}E_{1}^{2}}$$

Previous work used a different expression to calculate the carrier mobility (*μ*) of 2D materials [29,30]. Here, the following formula is used to estimate the relaxation time [30]:

(2) $$\mu^{2D}=\frac{2e\hbar^{3}C^{2D}}{3k_{B}T{\left|m^{*}\right|}^{2}E_{1}^{2}}$$

where *e* is the charge of electron, *ħ* is reduced Planck constant, *C*^3*D*^ is the 3D elastic constant, and *C*^2*D*^ is the 2D elastic modulus, which can be calculated through stress under uniaxial strain. *k_B_* is the Boltzmann constant, *T* is the absolute temperature, and m* is the effective mass of electrons and holes, which can be acquired by taking the second derivative of the electronic band structures. *E*_1_ is the deformation potential. The relaxation time of the carriers can be estimated by *τ* = μm*/e.

The electronic thermal conductivity is obtained according to the Wiedemann–Franz law as follows [31,32]:

(3) $$k_{e}=L\sigma T$$

Here, *L* is the Lorenz constant, which is set to 2.45 × 10^−8^ V^2^/K^2^ for black phosphorus and 1.5 × 10^−8^ V^2^/K^2^ for phosphorene, respectively; σ is the electrical conductivity; and *T* is the absolute temperature.

We employed the PHONOPY, version 2.15.1, [33] package to perform phonon dispersion calculations. The lattice thermal conductivity is solved using the ShengBTE package, version 1.2.0. [34]. The second-order force constants are calculated with supercell expansion factors of 3 × 3 × 3 and 5 × 5 × 1 for black phosphorus and phosphorene, respectively. The third-order force constants are calculated with supercell expansion factors of 3 × 2 × 3 and 4 × 4 × 1 for black phosphorus and phosphorene, respectively. The nearest neighbor atoms for the third-order force constant matrix are set to 12 for black phosphorus and phosphorene. To ensure the reliability of the computational results, the same calculation method is employed for phosphorene under tensile strain as that used for pristine phosphorene.

## 3. Results and Discussion

### 3.1. Crystal Structure and Electronic Properties

**Figure 1:**
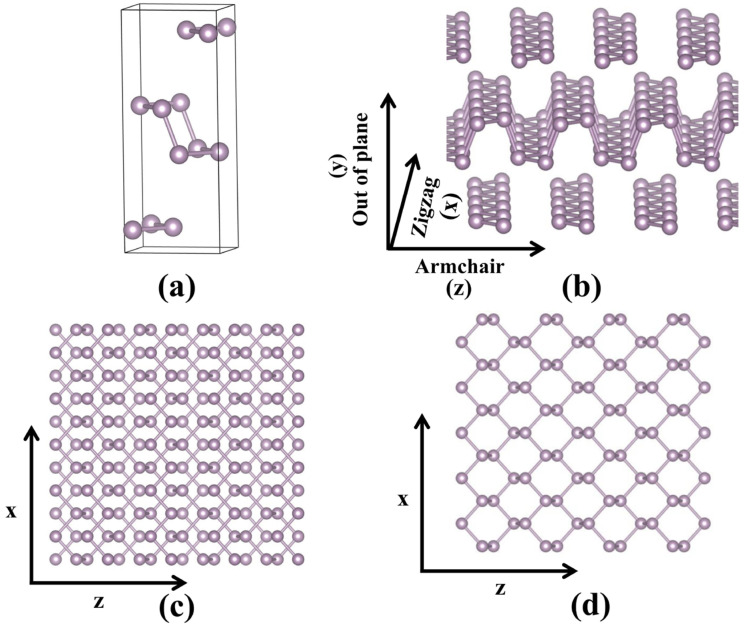
(**a**) The crystal structure, (**b**) side and (**c**) top views of the layer structure of bulk black-phosphorus, and (**d**) top view of monolayer phosphorene.

The crystal structures of bulk black phosphorus and monolayer phosphorene are shown in Figure 1. It is can be observed that both black phosphorus and phosphorene possess a layered, puckered honeycomb structure. The individual layers in black phosphorus are interconnected via Van der Waals forces. The optimized structural parameters of black phosphorus and phosphorene are presented in Table 1. The lattice parameters of bulk black phosphorus are a = 3.31 Å, b = 11.30 Å, and c = 4.56 Å, which are close to the experimental values [35]. It is can be seen that the in-plane lattice parameters of black phosphorus and phosphorene is obviously different. This phenomenon is consistent with previous studies [18,19,36] and can be attributed to Van der Waals interactions. In bulk black phosphorus, interlayer Van der Waals forces induce in-plane compressive strain. When exfoliated into a monolayer, this constraint is removed, causing an in-plane expansion for phosphorene that increases the lattice parameters along the armchair direction.

**Figure 2:**
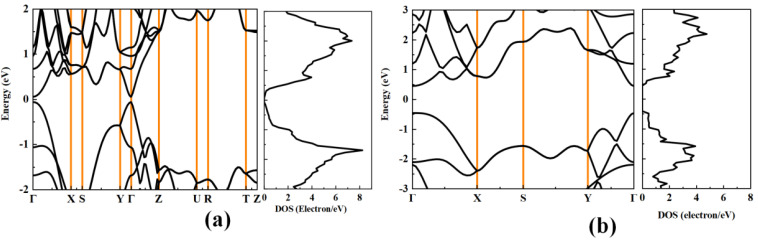
The calculated band structure and the corresponding DOS of (**a**) bulk black phosphorus and (**b**) black phosphorene.

Figure 2 shows the calculated band structure and density of states (DOS) of black phosphorus and phosphorene. The band gaps of black phosphorus and phosphorene are 0.12 eV and 0.91 eV, respectively, suggesting that black phosphorus could have larger electrical conductivity than phosphorene. It can be found that the bands near the valence band maximum (VBM) and conduction band minimum (CBM) disperse significantly along the out-of-plane direction (Γ–Y) and the armchair direction (Γ–Z), which means that black phosphorus possesses a small effective mass of electrons and holes. For phosphorene, the bands near VBM and CBM disperse significantly along the armchair direction (Γ–Y), which also indicates a very small effective mass of holes and electrons. Table 2 and Table 3 list the calculated carrier effective masses, elastic constant, deformation potential, and the electron relaxation time of black phosphorus and phosphorene. As we have analyzed from the band structure, the effective mass of black phosphorus along the Z direction is the smallest, and this is close to the results in Ref. [14] The DOS distributed around the Fermi level determines the magnitude of the Seebeck coefficient [37], the *p*-type black phosphorus and phosphorene may have a higher Seebeck coefficient than their *n*-type counterparts.

### 3.2. Transport Coefficients

**Figure 3:**
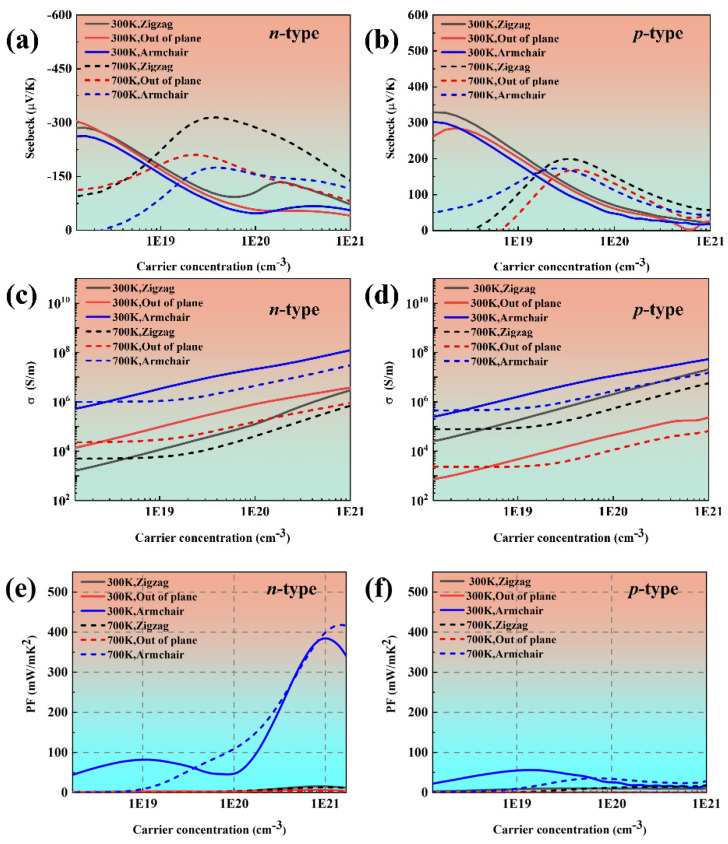
The transport property of black phosphorus as a function of carrier concentration at 300 K and 700 K: (**a**,**b**) the Seebeck coefficient, (**c**,**d**) electrical conductivity, and (**e**,**f**) power factor.

Figure 3 gives the calculated transport coefficients of phosphorus along the zigzag, out of plane, and armchair directions. It is can be observed that the in-plane Seebeck coefficient of black phosphorus exhibits a decreasing trend with an increasing charge carrier concentration that closely matches the results reported in Ref. [21]. Moreover, the Seebeck coefficient is close to the reported results in Ref. [38]. The Seebeck coefficient decreases along with the increased carrier concentration at 300 K when the carrier concentration is more than 1.5 × 10^18^ cm^−3^. This is because bulk black phosphorus enters the degenerate regime, and the Seebeck coefficient is proportional to $n^{-2/3}$  according to the formula $S=\frac{8\pi^{2}k_{B}^{2}}{3eh^{2}}m^{*}T{\left(\frac{\pi }{3n}\right)}^{\frac{2}{3}}$ [39]. It can be seen that the electrical conductivity of black phosphorus is larger than the reported results in Ref. [21]. This discrepancy may originate from the different computational methodologies employed for calculating the carrier relaxation time. Moreover, the electrical conductivity of black phosphorus along the armchair direction is always the highest at both 300 K and 700 K, which is consistent with our previous analysis of the effective mass and the reported results in Refs. [18,19,21]. The power factor of black phosphorus presents anisotropy owing to the anisotropic electrical conductivity.

**Figure 4:**
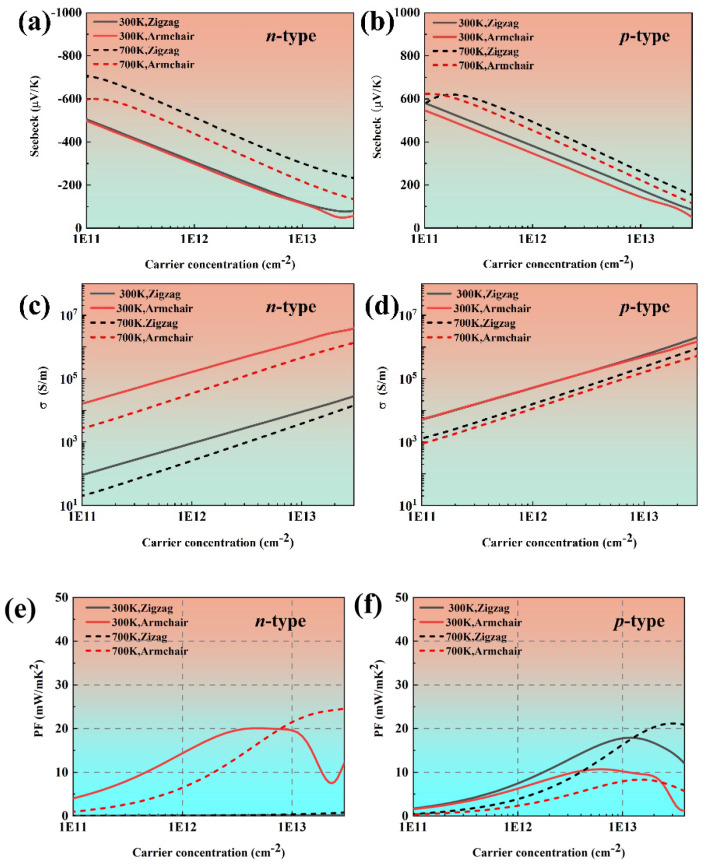
The electronic transport coefficients of phosphorene as a function of carrier concentration at 300 K and 700 K: (**a**,**b**) the Seebeck coefficient, (**c,d**) electrical conductivity, and (**e**,**f**) power factor.

Figure 4 demonstrates the calculated transport coefficients along the zigzag and armchair directions. It is can be clearly observed that along both the armchair and zigzag directions, *p*-type phosphorene consistently exhibits higher absolute values of the Seebeck coefficient compared with its n-type counterpart, which is full agreement with Ref. [36]. Moreover, the Seebeck coefficient of phosphorene is larger than that of black phosphorus owing to the quantum confinement effect. In bulk black phosphorus, charge carriers can originally move freely in 3D space. However, due to the atomic-scale thickness of monolayer phosphorene, the carriers are strongly confined within a 2D plane, thereby giving rise to quantum confinement effects. This modifies the band structure and significantly increases the DOS of electrons near the Fermi level. Thus, the Seebeck coefficient is enhanced. Moreover, the Seebeck coefficient exhibits different behaviors for *n*- and *p*-type phosphorene due to the anisotropic crystal structure. It can be seen that the electrical conductivity of phosphorene is anisotropic, whereas the *p*-type phosphorene remains nearly isotropic at both 300 K and 700 K. We attribute this phenomenon to the multi-valley structure near CBM. At the same time, the electrical conductivity of *n*-type and *p*-type phosphorene is always the highest along the armchair direction, which is consistent with our previous analysis of effective mass. The power factor of black phosphorene also presents anisotropy. Moreover, due to the high electrical conductivity, the power factor of *n*-type phosphorene is significantly higher than that of *p*-type phosphorene. The power factor of *n*-type phosphorene can reach up to 18.92 mW/mK^2^, corresponding to a carrier concentration of −4.97 × 10^12^ cm^−2^, which is higher than that of most conventional TE materials.

**Figure 5:**
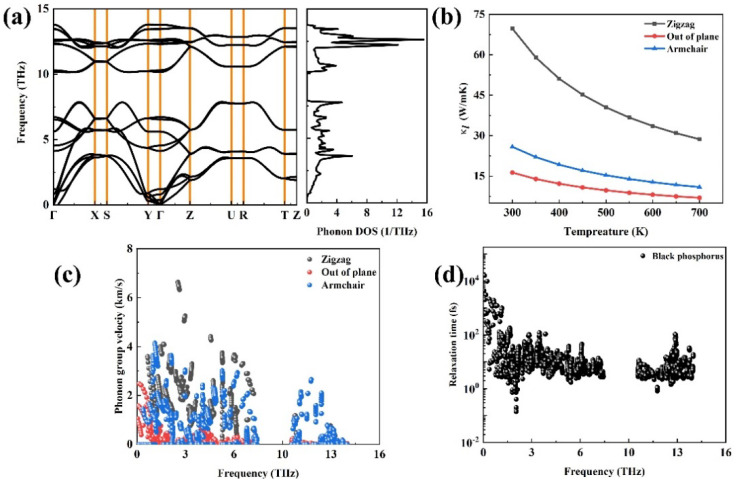
The phonon transport property of black phosphorus: (**a**) the phonon dispersion relations and phonon DOS, (**b**) the calculated lattice thermal conductivity as a function of the temperature of black phosphorus along three directions, (**c**) the phonon group velocity, and (**d**) the phonon relaxation time.

Figure 5a plots the phonon dispersion relation of black phosphorus, and it can be seen that there is no imaginary frequency in black phosphorus suggesting that this structure is dynamically stable. Among the three acoustic phonon modes, it can be seen that the dispersion is the largest along the Γ–X direction, whereas it is the smallest along the Γ–Y direction. It is moderate along the Γ–Z direction. At 300 K, the lattice thermal conductivity is 69.7 W/mK^2^ along the zigzag direction and 25.84 W/mK^2^ along the armchair direction, and these findings are close to the values in Ref. [20]. To elucidate this phenomenon, we further calculated the phonon group velocities of black phosphorus along three directions in Figure 5c. The relationship of the phonon group velocity is as follows: *V_Γ–X_* > *V_Γ–Z_* > *V_Γ–Y_*.

**Figure 6:**
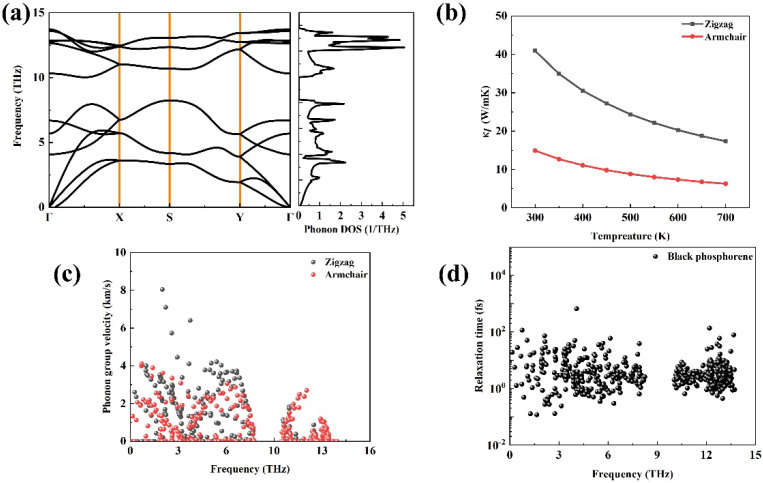
The phonon transport property of phosphorene: (**a**) the phonon dispersion relations and phonon DOS, (**b**) the calculated lattice thermal conductivity as a function of the temperature of phosphorene along two directions, (**c**) the phonon group velocity, and (**d**) the phonon relaxation time.

Figure 6a plots the phonon dispersion relations of phosphorene, and it can be found that there is no imaginary frequency for phosphorene, which suggests that this structure is dynamically stable. Between the two acoustic phonon modes, it can be seen that the dispersion along the Γ–X direction is obviously larger than that along the Γ–Y direction. At 300 K, the lattice thermal conductivity is 40.92 W/mK^2^ along the zigzag direction and 14.9 W/mK^2^ along the armchair direction, respectively, which is close to the results in Ref. [40]. Figure 5c exhibits the phonon group velocity along two directions. Similar to the phonon vibration frequency, the phonon group velocity along the Γ–X direction is also the largest.

### 3.3. Thermoelectric Figure of Merit

**Figure 7:**
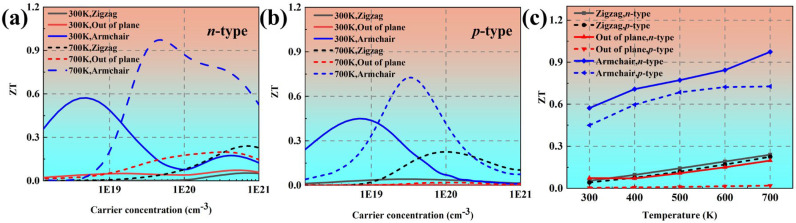
The calculated *ZT* values of black phosphorus as a function of carrier concentration at (**a**) 300 K and (**b**) 700 K. (**c**) The optimal *ZT* values of black phosphorus as a function of temperature.

Figure 7a,b show the *ZT* values as a function of the carrier concentration at 300 K and 700 K, respectively. The power factor along the Z direction is relatively much larger, which leads to an obviously larger *ZT* value along the Z direction. At the optical electron concentration of 4.92 × 10^18^ cm^−3^, the *ZT* value along the armchair direction can reach up to 0.59 at 300 K. At 700 K, the *ZT* value is improved to 0.97. Figure 7c plots the temperature-dependent optimized *ZT* values. It is evident that the *ZT* value exhibits a monotonic increase with increased temperature in all three directions, primarily attributed to the enhancement of the Seebeck coefficient and the reduction in lattice thermal conductivity.

**Figure 8:**
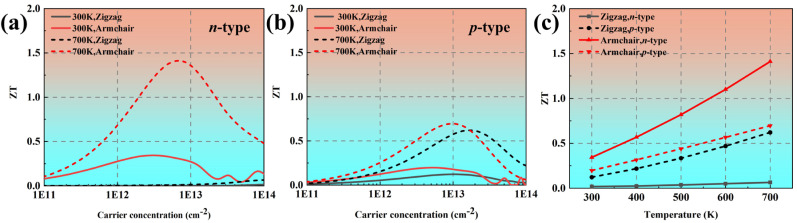
The calculated *ZT* values of phosphorene as a function of carrier concentration at (**a**) 300 K and (**b**) 700 K. (**c**) The optimized *ZT* values of phosphorene as a function of temperature.

Figure 8a,b show the *ZT* values of phosphorene as a function of the carrier concentration at 300 K and 700 K, respectively. The *ZT* value of *n*-type phosphorene along the armchair direction is 0.34, which is between the reported results in Refs. [18,36]. This indicates that our calculated *ZT* value of phosphorene is reasonably. The highest *ZT* value arrived at 1.41, corresponding to electron concentration of 5.53 × 10^12^ cm^−2^ along the Z direction at 700 K, which is 0.45 times higher than that of black phosphorus (*ZT* = 0.97). Figure 8c shows the temperature-dependent *ZT* values. It can be seen that the isotropic *ZT* value of *n*-type phosphorene significantly increases with increasing temperature, while the isotropy of *p*-type phosphorene decreases with increasing temperature.

### 3.4. Further Optimized ZT Values of Phosphorene

**Figure 9:**
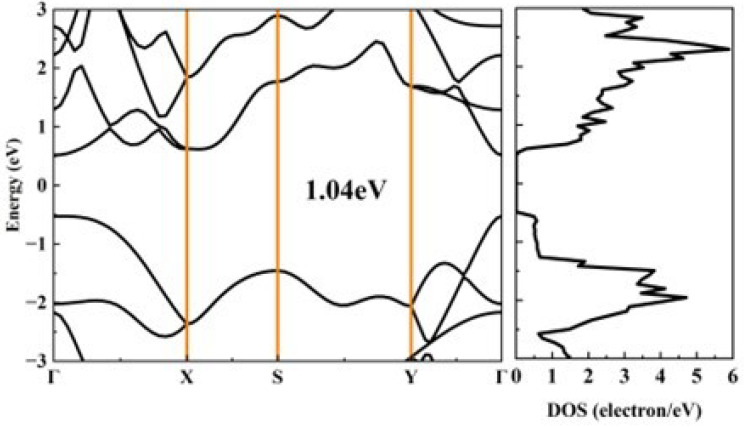
The calculated band structure and the corresponding DOS of phosphorene under 4.5% tensile strain along the armchair direction.

**Figure 10:**
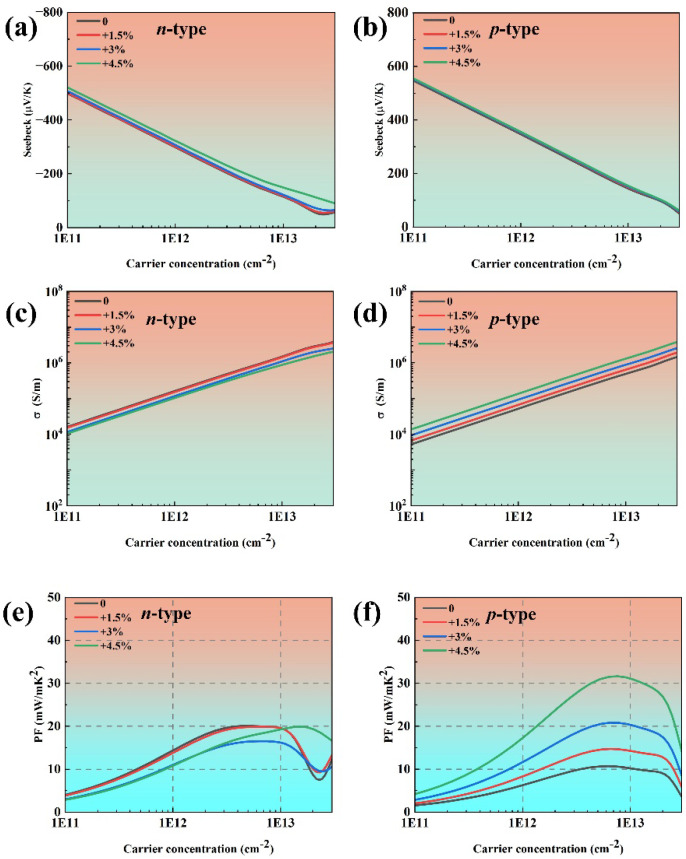
The electronic transport coefficients of phosphorene under tensile strain along the armchair direction ranging from 0% to 4.5% as a function of carrier concentration: (**a**,**b**) the Seebeck coefficients at 300 K, (**c**,**d**) the electrical conductivities at 300 K, and (**e**,**f**) the power factors at 300 K.

In 2024, Habiba Mamori et al. found that the TE performance of bilayer phosphorene could be enhanced under 3% uniaxial tensile strain along the armchair direction, and the *ZT* value of *n*-type bilayer phosphorene could be improved by 110% compared with the *n*-type pristine bilayer phosphorene at 300 K [41]. Therefore, we attempt to enhance the TE performance of phosphorene by applying uniaxial tensile strain along the armchair direction. Figure 9 illustrates the calculated band structure and the corresponding DOS of phosphorene under 4.5% tensile strain along the armchair direction. The band gap of phosphorene increases from 0.91 eV to 1.04 eV under 4.5% tensile strain. The DOS near the Fermi level under 4.5% tensile strain is similar to that of pristine phosphorene, indicating a comparable Seebeck coefficient, as shown in Figure 10a,b. As the TE performance of phosphorene is best along the armchair direction, we focus on the TE transport properties of phosphorene along the same direction. First, applying strain along the armchair direction has almost no effect on the effective mass of phosphorene, which is consistent with the findings reported in Ref. [42]. Second, the deformation potential of electrons increases with the augmentation of strain, while the deformation potential of holes decreases with the augmentation of strain. This phenomenon could be attributed to the modified crystal structure of phosphorene. The VBM, dominated by *p_z_* orbitals (Figure S1), exhibits reduced interatomic orbital overlap of *p_z_* by bond-length elongation because of tensile strain, which could consequently weaken electron–phonon coupling strength. The reduced influence of lattice vibration scattering on charge carriers at the VBM leads to a corresponding decrease in the deformation potential energy. Third, the elastic constant decreases with increased tensile strain as the enlarged lattice constant along the armchair direction weakens interactions between phosphorus atoms. From Equation (1), it can be inferred that the deformation potential has a more significant contribution to the relaxation time compared with the elastic constants. Thus, the relaxation time of holes increases with increasing strain, which is beneficial for electrical conductivity. When a 4.5% tensile strain is applied to phosphorene along the armchair direction, the maximum power factor increases from 10.69 mW/mK^2^ to 31.63 mW/mK^2^ for *p*-type phosphorene at room temperature, as shown in Figure 10d.

**Figure 11:**
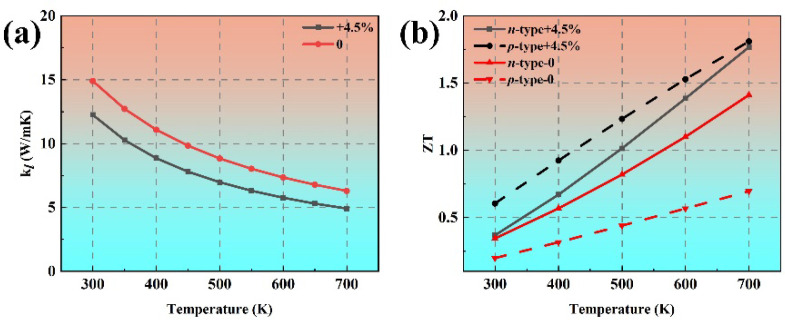
Comparison of the (**a**) temperature-dependent lattice thermal conductivity and (**b**) *ZT* values of pristine phosphorene and phosphorene under 4.5% tensile strain along the armchair direction.

Figure 11a plots the lattice thermal conductivity of phosphorene under 4.5% tensile strain along the armchair direction as a function of temperature from 300 K to 700 K. At room temperature, the calculated thermal conductivity of phosphorene is 12.25 W/mK under 4.5% tensile strain along the armchair direction, which is 16% smaller than that of pristine phosphorene. It can be found that the electronic transport property of *p*-type phosphorene can be enhanced obviously by increasing the relaxation time of holes under the 4.5% tensile strain along the armchair direction. The *ZT* value of *p*-type phosphorene can reach up to 0.60 at 300 K under 4.5% tensile strain along the armchair direction, as shown in Figure 11b, which is much larger than that of pristine *p*-type black phosphorene (*ZT* = 0.20). Moreover, for phosphorene under 4.5% tensile strain along the armchair direction, the *ZT* values for both *p*- and *n*-type systems exceed 1.7 at 700 K, which is significantly larger than that of pristine phosphorene (*n*-type *ZT* = 1.41, *p*-type *ZT* = 0.70). These *ZT* values are obviously larger than bilayer phosphorene [41], monolayer SnS and PbS [43], and MoSe_2_/WSe_2_ superlattice heterostructures [44]. Our calculations suggest that the TE performance of phosphorene can be further enhanced under tensile strain along the armchair direction. Moreover, phosphorene under tensile strain not only exhibits exceptional TE performance but also avoids the use of rare, expensive, or toxic heavy metals. Therefore, phosphorene under tensile strain can be used with high efficiency in non-toxic, metal-free, low-cost, and ultra-light TE devices.

## 4. Summary

The TE properties of bulk black phosphorus without strain and phosphorene under tensile strain along the armchair direction ranging from 0% to 4.5% are investigated using the first-principles calculations and Boltzmann transport theory with relaxation time approximation. Compared with black phosphorus, phosphorene has obviously larger Seebeck coefficient owing to the quantum confinement effect. Phosphorene has lower lattice thermal conductivity than black phosphorus because of dimensional effects, changes in phonon DOS, and lower phonon relaxation time. At 700 K, black phosphorus and phosphorene are found to have large *ZT* values of 0.97 and 1.41, respectively. After applying 4.5% tensile strain along the armchair direction, the electrical conductivity of *p*-type phosphorene is enhanced because of the increased hole relaxation time. Moreover, the tensile strain also decreases the lattice thermal conductivity. Thus, the *ZT* values for both *p*- and *n*-type phosphorene exceed 1.7 at 700 K. Our theoretical work suggests that TE performance can be improved by identifying low-dimensional systems or applying tensile strain. In addition, phosphorene under tensile strain can be used at high efficiency in non-toxic, metal-free, low-cost, and ultra-light TE devices.

## Figures and Tables

**Table 1:** Structural parameters of bulk black phosphorus and phosphorene.

Materials	Reference	a (Å)	b (Å)	c (Å)
Phosphorus	Experiment [35]	3.31	10.47	4.37
	This work	3.31	11.30	4.56
Phosphorene	This work	3.30		4.62

**Table 2:** Calculated carrier effective mass (m*), elastic modulus (*C*^3*D*^), deformation potential constant (*E*_1_), and relaxation time (*τ*) of electrons and holes for black phosphorus along the zigzag, armchair, and out of plane directions at 300 K.

Carrier Type	m* (*m_e_*)	*C^3D^* (*J/m*^3^)	*|E*_1_*|* (*eV*)	τ(*fs*)
e (Zigzag)	1.27	87.90	5.91	61.32
h (Zigzag)	1.42	87.90	1.36	979.44
e (Armchair)	0.16	21.23	2.91	1332.75
h (Armchair)	0.15	21.23	4.78	582.58
e (Out of plane)	0.21	6.58	7.05	47.72
h (Out of plane)	0.44	6.58	9.20	9.29

**Table 3:** Calculated carrier effective mass (m*), in-plane elastic modulus (*C*^2*D*^), deformation potential constant (*E*_1_), and relaxation time (*τ*) of electrons and holes for phosphorene along the zigzag and armchair directions at 300 K.

Carrier Type	m* (*m_e_*)	*C*^2*D*^ (*J/m*^2^)	*|E*_1_*|* (*eV*)	τ(*fs*)
e (Zigzag)	1.24	51.56	5.29	11.99
h (Zigzag)	2.94	51.56	0.28	1735.37
e (Armchair)	0.18	12.18	1.44	261.83
h (Armchair)	0.17	12.18	2.7	80.54

## Data Availability

The original contributions presented in this study are included in the article/Supplementary Materials. Further inquiries can be directed to the corresponding authors.

## Supplementary Materials

The following supporting information can be downloaded at: https://www.mdpi.com/article/10.3390/ma18112506/s1, Figure S1: Calculated partial DOS of phosphorene with and without tensile strain; Table S1: Calculated carrier effective mass (*m^∗^*), in-plane elastic modulus (*C^2D^*), deformation potential constant (*E_1_*), and electron relaxation time (*τ*) of phosphorene under tensile strain along the armchair direction at 300 K.
