# Supplementary material for: Enhanced Thermoelectric Properties of Phosphorene via Quantum Size Effects and Relaxation Time Tuning
Source: Materials (Basel). 2025 May 26;18(11):2506. doi: 10.3390/ma18112506 (PMC12156281; doi:10.3390/ma18112506)
Supplement: Supplementary file 1 [file materials-18-02506-s001.zip › materials-3622919-supplementary.pdf]

Supplement Information

Enhanced Thermoelectric Properties of Phosphorene by Quantum Size Effect and Relaxation Time Tuning

Zhiqian Sun[a, 1], Chenkai Zhang[a, 1], Guixian Ge[a], Gui Yang[b*], Jueming Yang[a*]

[a]College of Sciences/State Key Laboratory of Advanced Energy Storage Materials and Technology, Shihezi University, Shihezi City 832000, China

[b]School of Mechanical and Electrical Engineering，Chuzhou University, Chuzhou 239000, P. R. China

[*]Corresponding authors: guixiange@shzu.edu.cn (G. Ge); kuiziyang@chzu.edu.cn (G. Yang); juemingyang@shzu.edu.cn (J. Yang).

1 These authors contributed equally to this work.

Supplement Information

Table S1. Calculated carrier effective mass (m^*), in-plane elastic modulus (C^{2D}), deformation potential constant (E_l), and electron relaxation time (τ) of phosphorene under tensile strain along the armchair direction at 300 K.

Materials	*Carrier type*	$m^*(m_e)$	$C^{2D}(J/m^2)$	$\|E_l\|(eV)$	$\tau(fs)$
BP(1.5%)	e (armchair)	0.179	11.77	1.46	250.41
	h (armchair)	0.168	11.77	2.29	107.80
BP(3%)	e (armchair)	0.178	11.43	1.64	193.38
	h (armchair)	0.168	11.43	1.93	146.85
BP(4.5%)	e (armchair)	0.175	11.01	1.61	195.01
	h (armchair)	0.168	11.01	1.55	220.98

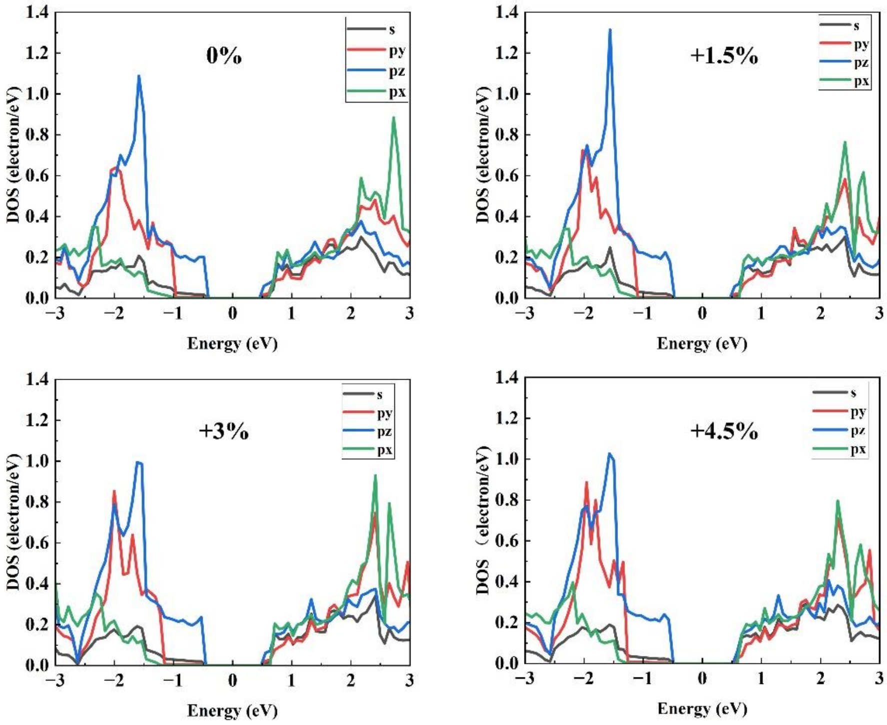

Figure S1. Calculated partial DOS of phosphorene with and without tensile strain.
